# Clinical impact of Wnt5a expression on persistence of acute kidney injury in patients with urosepsis

**DOI:** 10.1080/0886022X.2024.2369176

**Published:** 2024-06-24

**Authors:** Jungho Shin, Yoosik Yoon, Dong-Jin Oh

**Affiliations:** a Department of Internal Medicine, Chung-Ang University College of Medicine, Seoul, South Korea; b Department of Microbiology, Chung-Ang University College of Medicine, Seoul, South Korea; c Department of Internal Medicine, Myongji Hospital, Hanyang University College of Medicine, Goyang, South Korea

**Keywords:** Acute kidney injury, major adverse kidney event, renal recovery, sepsis, Wnt5a

## Abstract

Abnormal Wnt5a expression is associated with dysregulated inflammation and organ dysfunction. However, the effect of Wnt5a activation on the duration of organ dysfunction remains unclear. This prospective study investigated the association between Wnt5a levels and persistent acute kidney injury (AKI) in patients with urosepsis. Serum creatinine and Wnt5a levels were measured on days 1 and 5 and at discharge in 87 patients diagnosed with urosepsis. Patients with urosepsis were classified into an improving acute kidney injury (AKI) group and a persistent or worsening AKI group according to the AKI stage on days 1 and 5. AKI recovery was defined as a discharge-to-baseline serum creatinine ratio of <1.5. Twenty-eight patients with urosepsis (32.2%) had persistent or worsening AKI, and their Wnt5a levels were higher on days 1 and 5 and at discharge than those with improving AKI. The association between Wnt5a levels and persistent or worsening AKI was maintained after adjusting for age, sex, baseline serum creatinine levels, and disease severity. Moreover, elevated Wnt5a levels were associated with an increased risk of major adverse kidney events. High Wnt5a levels at discharge were associated with unrecovered AKI and participants with AKI recovery had a steeper Wnt5a slope over time than those without recovery, irrespective of age, sex, baseline serum creatinine level, or disease severity. Assessment of Wnt5a expression was helpful in predicting AKI persistence and adverse outcomes in patients with urosepsis. Therefore, Wnt5a may serve as a valuable bio-marker for identifying the risk of persistence of AKI.

## Introduction

Sepsis is a serious condition that leads to life-threatening organ dysfunction resulting from a dysregulated response to infection [1]; a dysregulated inflammatory response is responsible for organ dysfunction and poor outcomes. Acute kidney injury (AKI) is a common complication of sepsis, with occurrence rate varying between 25 and 75% [2]. Up to a quarter of patients with sepsis also have AKI, require renal replacement therapy (RRT), and have the worst prognosis [3,4]. After AKI onset, diverse reversal patterns take place. In one analysis of 16,968 critically ill patients with moderate to severe AKI, five patterns were described: early sustained reversal (25.6%); late sustained reversal (9.7%); relapse, recovery (22.5%); relapse, no recovery (14.7%); and never reversed (26.5%) [5]. Several patients do not exhibit reversal after AKI, and those without early reversal after AKI experience reduced survival compared to patients with early reversal [5]. The prognosis of sepsis-associated AKI is determined by its severity and recovery pattern; those with partial recovery—defined as improved but with a last known serum creatinine to the reference creatinine ratio ≥1.5—seem to have similar prognoses to those without AKI [6]. Given the clinical importance of AKI persistence and reversal pattern, the Acute Disease Quality Initiative (ADQI) workgroup has proposed the consensus definition of persistent AKI and acute kidney disease: sustained AKI beyond 48 h from its onset; and acute or subacute damage and/or loss of kidney function for a duration of between 7 and 90 days after AKI event [7]. Therefore, recognizing the risk of persistent AKI and estimating reversibility is fundamental for optimizing therapeutic strategies for patients with high AKI risk, such as those with sepsis.

The Wnt ligand family comprises secreted glycoproteins that play an essential role in embryonic development and tissue homoeostasis by activating an evolutionarily conserved pathway. Thus, any aberration in Wnt signaling can lead to pathology, and research has revealed its involvement in various human diseases [8]. Non-canonical Wnt signaling is linked to inflammatory conditions such as rheumatoid arthritis, psoriasis, atherosclerosis, and sepsis [9–11]. Wnt5a, an extensively studied non-canonical Wnt ligand, has been shown to play an important role in inducing an inflammatory phenotype by up-regulating genes encoding pro-inflammatory cytokines and activating pro-inflammatory pathways, such as nuclear factor-κB [12,13]. The role of Wnt5a in sepsis has also been investigated, and it is reportedly elevated in patients with sepsis. Moreover, high Wnt5a levels indicate severe disease and the development of adverse outcomes [14–17]. However, there is a literature gap regarding the extensive role of this protein in sepsis. It also remains unclear whether Wnt5a expression is associated with persistent organ injury, despite the established link between Wnt5a and inflammation.

In this prospective study, we investigated the association between Wnt5a expression and the persistence of kidney disease in patients with urosepsis, as the kidneys are frequently involved in sepsis. The relationship of Wnt5a expression with AKI duration and reversal pattern was assessed using serial Wnt5a measurements throughout the study period. The effect of Wnt5a on disease severity was also determined by assessing the incidence of major adverse kidney events (MAKEs). Accordingly, this study comprehensively evaluated the potential of Wnt5a as a bio-marker for predicting outcomes in individuals with urosepsis.

## Materials and methods

### Study design and subjects

**Figure 1.**
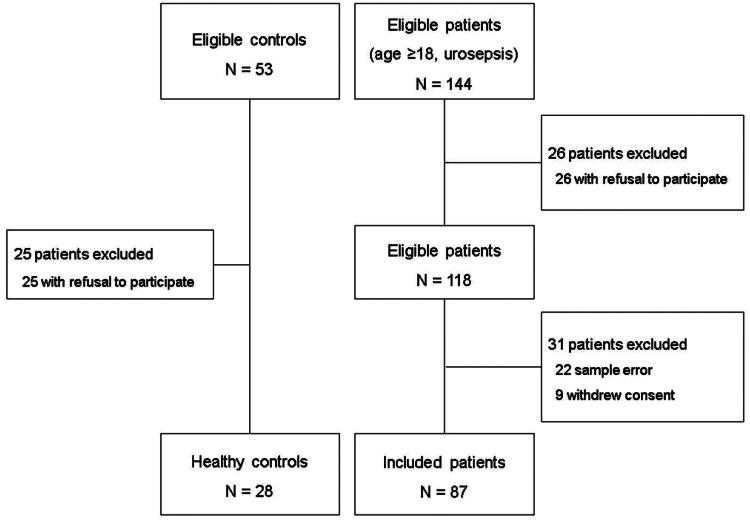
Study flow chart of subject inclusion. Individuals who visited the hospital for health screening were requested for the study inclusion to the control group. On the other hand, adult (age ≥18) patients with urosepsis who admitted to the intensive care unit were eligible for the patient group. Written informed consent was obtained from each participant or his or her legal representative before the study inclusion.

This prospective observational investigation was designed as a pilot study and was approved by the institutional ethics committee of the hospital (number: MJH 2020-09-002). Before inclusion, written informed consent was obtained from each participant or their legal representative. This study recruited adult patients (>18 years old) with urinary tract infection and sepsis who were admitted to Myongji Hospital between January 2018 and April 2023. For inclusion, the researchers confirmed the absence of the exclusion criteria, which included patients with end-stage kidney disease undergoing dialysis and those with urinary tract obstruction requiring urinary diversion. Namely, patients with chronic kidney disease were not excluded if they did not require RRT. Urinary tract infection was defined as pyuria on urinalysis or the presence of a urine culture-confirmed pathogen. Sepsis was defined according to the Third International Consensus Definitions for Sepsis and Septic Shock [17]. Healthy volunteers were also recruited as controls, were screened during the general health checkup, and were not matched to participants of the patient group. Figure 1 presents a flow chart of participants inclusion in this study.

### Data collection

Baseline demographic and clinical data of patients with urosepsis were collected on the admission day. Disease severity was estimated according to the Sequential Organ Failure Assessment (SOFA) score [17]. Serum creatinine and Wnt5a levels were measured on days 1 and 5 and at discharge. Samples were collected at discharge from deceased participants; however, some measurements were missing for these participants (two day-5 samples and four discharge-day samples). Missing values were replaced with the last measured values. Baseline renal function was determined using nadir creatinine levels between 7 days and 24 months before admission. If available serum creatinine measurements were absent (*n* = 46 [52.9%]), baseline creatinine levels were computed by back-calculation using the Modification of Diet in Renal Disease equation, with reference values for age and sex [18]. In healthy controls, the data collected included age, sex, and serum creatinine levels. The estimated glomerular filtration rate (eGFR) was calculated using the Modification of Diet in Renal Disease equation [19].

For the measurements of Wnt5a, samples were centrifuged at 1000 *g* for 20 min and were aliquoted and stored at −80 °C before analysis. Wnt5a protein levels were evaluated using an enzyme-linked immunosorbent assay (ABclonal Biotechnology, Woburn, MA, USA). The assays were performed according to the manufacturer’s instructions.

### Definitions and outcomes

AKI presence and severity was identified according to the Kidney Disease: Improving Global Outcomes clinical practice guidelines for AKI [20]: stage 1 was defined as a day 1/baseline serum creatinine ratio of 1.5–1.9; stage 2, 2.0–2.9; and stage 3, ≥3.0. Subsequently, the AKI stage was assessed by the same method using the day 5/baseline ratio. If participants underwent RRT when measuring serum creatinine or died, they were classified as stage 3. Patients were divided into improving and persistent or worsening AKI groups according to the AKI stage on days 1 and 5.

The incidence of MAKEs, defined as stage 3 AKI, RRT initiation, or 30-day death, was evaluated. This study further evaluated the composite outcomes, including RRT need and mortality, and RRT- and intensive care unit (ICU)-free days over 30 days.

The AKI reversal patterns were evaluated. AKI recovery was defined as a discharge/baseline serum creatinine ratio of <1.5, while unrecovered AKI was defined as a ratio of ≥1.5 or death. Trajectories of Wnt5a levels over time were compared according to the reversal pattern.

### Statistical analysis

We employed the Kolmogorov-Smirnov test and Quantile-Quantile plot to assess the normality of continuous variables. Continuous variables are expressed as medians (inter-quartile range) and compared using the Mann–Whitney U test, while categorical variables are expressed as numbers (percentages) and compared using the chi-square test. The correlation between Wnt5a and measured variables was evaluated using Spearman’s rank correlation coefficient. Logistic regression analysis was performed to determine the probability of clinical outcomes, including persistent or worsening AKI, MAKE and its components, and AKI recovery, which were expressed as odds ratio (ORs). The fit of logistic regression analysis was confirmed using the Hosmer-Lemeshow test. The receiver operating characteristic (ROC) curve for Wnt5a expression was derived to predict persistent or worsening AKI. Additionally, the cumulative incidence of composite outcomes, comprising RRT initiation or death over 30 days, was compared using the Kaplan–Meier test with the log-rank test and Cox regression analysis. The effect of Wnt5a levels on RRT- and ICU-free days was assessed using linear regression analysis. Moreover, the slope of the change in Wnt5a over time was estimated using the linear mixed-effect model, permitting missing values of Wnt5a, and compared according to the reversal pattern after AKI. All multivariate analyses were adjusted for age, sex, baseline serum creatinine level, and SOFA score. All statistical analyses were performed using SPSS Statistics software (version 26.0; IBM Corp., Armonk, NY, USA). Statistical significance was set at *p* < 0.05.

## Results

### Comparison of Wnt5a levels between healthy individuals and patients with urosepsis

This study included 28 healthy individuals and 87 patients with urosepsis (Figure 1). Age, percentage of males, and eGFR were 42 (35–46) years, 11 (39.3%), and 98.2 (93.5–107.6) mL/min/1.73 m^2^, respectively, in the control group, while the corresponding values were 80 (69–85) years, 21 (24.1%), and 66.0 (62.0–89.8) mL/min/1.73 m^2^, respectively, in the patient group (*p* < 0.001, *p* = 0.120, and *p* < 0.001, respectively). Wnt5a levels differed between healthy individuals and patients with urosepsis (1.8 [1.4–2.4] ng/mL versus 2.1 [1.8–2.5] ng/mL; *p* = 0.023). No correlation was noted between Wnt5a expression and age, sex, and eGFR in healthy participants (*p* = 0.417, 0.663, and 0.434, respectively).

### Characteristics of patients with urosepsis according to AKI persistence

Among patients with urosepsis, 58 (66.7%) and 29 (33.3%) were classified into the improving and persistent or worsening AKI groups, respectively (Table 1). Age, percentage of males, comorbidities, and baseline eGFR did not differ between the two groups. In contrast, levels of platelets and albumin were lower, and outcome parameters, such as SOFA score, RRT requirement and duration, ICU duration, and mortality, were poorer in patients of the persistent or worsening AKI group compared to those of the improving AKI group. Wnt5a levels were higher in the persistent or worsening AKI group than in the improving AKI group (*p* = 0.012, 0.009, and 0.001, respectively). Correlation analysis revealed no association between Wnt5a levels and age, sex, comorbidities, eGFR, or laboratory parameters. Additionally, Wnt5a levels were not related to SOFA score, AKI stage, or RRT in patients with urosepsis.

**Table 1.** Baseline characteristics of patients with urosepsis according to the persistence of AKI.

	Improving AKI (*N* = 58)	Persistent or worsening AKI (*N* = 29)	*P*
Age, years	80 (70–84)	80 (69–85)	0.892
Male, *n* (%)	11 (19.0)	10 (34.5)	0.111
Comorbidities, *n* (%)			
Hypertension	38 (65.5)	20 (69.0)	0.748
Diabetes	32 (56.2)	19 (65.5)	0.356
Chronic kidney disease	7 (12.1)	6 (20.7)	0.288
Heart failure	4 (6.9)	4 (13.8)	0.432
Coronary artery disease	7 (12.1)	6 (20.7)	0.288
Chronic lung disease	2 (3.4)	0 (0.0)	0.550
Chronic liver disease	2 (3.4)	0 (0.0)	0.550
Malignancy	3 (5.3)	3 (10.7)	0.391
Baseline creatinine, mg/dL	0.9 (0.8–0.9)	0.9 (0.6–1.1)	0.324
Baseline eGFR, mL/min/1.73 m^2^	65.5 (62.0–86.7)	70.0 (61.0–102.3)	0.549
Laboratory results			
White blood cell, /mm^3^	1450 (1013–2168)	1310 (605–2045)	0.235
Haemoglobin, g/dL	10.7 (9.4–12.2)	10.2 (8.6–11.8)	0.245
Platelets, /mm^3^	194 (133–241)	127 (69–205)	0.013
Albumin, g/dL	3.5 (3.0–3.9)	3.3 (2.8–3.6)	0.089
C-reactive protein, mg/dL	14.7 (7.5–21.1)	17.2 (9.0–20.9)	0.529
Lactate, mmol/L	3.0 (1.8–5.4)	3.3 (1.5–5.3)	0.842
SOFA score	6 (5–8)	7 (5–10)	0.095
Septic shock, *n* (%)	43 (74.1)	23 (79.3)	0.595
AKI stage, *n* (%)			0.095
No AKI	21 (36.2)	4 (13.8)	
Stage 1	9 (15.5)	3 (10.3)	
Stage 2	11 (19.0)	8 (27.6)	
Stage 3	17 (29.3)	14 (48.3)	
RRT requirement, *n* (%)	4 (6.9)	15 (51.7)	<0.001
RRT duration, days	0 (0, 0)	1 (0, 11)	<0.001
ICU duration, days	3 (2, 5)	8 (3, 12)	<0.001
30-day death, *n* (%)	3 (5.2)	8 (27.6)	0.005
Wnt5a, ng/mL			
On day 1	2.0 (1.8–2.3)	2.3 (2.0–2.6)	0.012
On day 5	1.9 (1.7–2.3)	2.3 (1.9–2.7)	0.009
At discharge	2.0 (1.7–2.3)	2.5 (2.1–2.7)	0.001

Data are expressed as median (interquartile range) or number (%).

AKI: acute kidney injury; ICU: intensive care unit; RRT: renal replacement therapy; SOFA: Sequential Organ Failure Assessment.

Improving AKI refers to a reduced stage of AKI from days 1 to 5, and persistent or worsening AKI refers to a maintained or elevated stage of AKI from days 1 to 5.

**Table 2.** The ORs of Wnt5a for persistent or worsening AKI in patients with urosepsis.

	Univariate		Multivariate^a^	
Variable	OR (95% CI)	*P*	OR (95% CI)	*P*
Wnt5a on day1	2.5 (1.0–5.8)	0.041	2.5 (1.0–5.9)	0.044
Wnt5a on day 5	4.4 (1.5–12.5)	0.005	4.9 (1.6–14.7)	0.005
Wnt5a on discharge day	6.4 (2.1–19.5)	0.001	7.5 (2.4–24.0)	0.001

AKI: acute kidney injury; CI: confidence interval; OR: odds ratio.

Improving AKI refers to a reduced stage of AKI from days 1 to 5, and persistent or worsening AKI refers to the same or an elevated stage of AKI from days 1 to 5.

^a^ Adjusted for age, sex, baseline serum creatinine level, and sequential organ failure assessment score.

**Figure 2.**
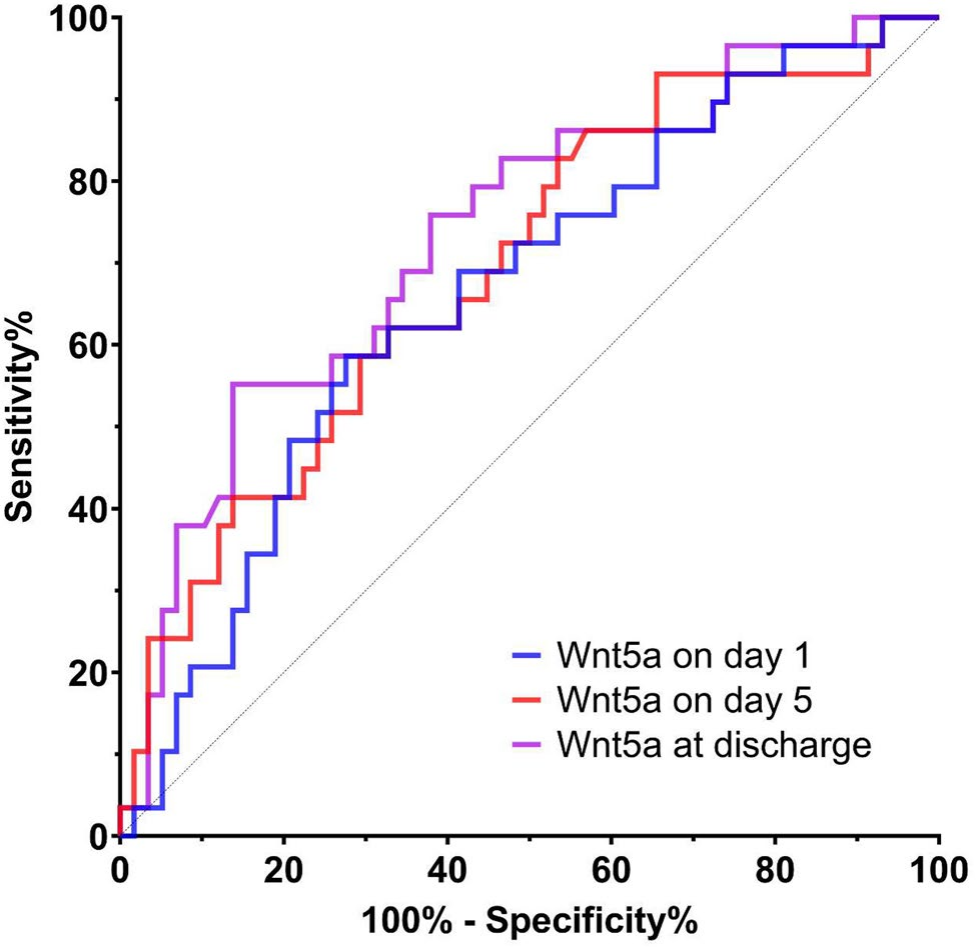
The receiver operating characteristic curve for persistence of AKI according to Wnt5a value. All measurements of Wnt5a predicted the persistent or worsening AKI in patients with urosepsis: the area under the curve (AUC) of Wnt5a on day 1 was 0.66 (95% CI 0.54–0.78; *p* = 0.014); the AUC of Wnt5a on day 5, 0.69 (95% CI 0.57–0.81; *p* = 0.004); and the AUC of Wnt5a on discharge day, 0.74 (95% CI 0.62–0.85; *p* < 0.001). AKI: acute kidney injury; CI: confidence interval. Improving AKI refers to a reduced stage of AKI from days 1 to day 5, and persistent or worsening AKI refers to a maintained or elevated stage of AKI from days 1 to 5.

We also calculated the probability of persistent or worsening AKI according to Wnt5a levels, represented as OR (Table 2). All Wnt5a measurements showed a consistent association with persistent or worsening AKI irrespective of age, sex, baseline serum creatinine level, or SOFA score. We further illustrated the ROC curves and determined the potential of Wnt5a levels for predicting persistent or worsening AKI in patients with urosepsis (Figure 2). Based on the results, Wnt5a measurements predicted the occurrence of persisting or worsening AKI in patients with urosepsis.

### MAKE, stage 3 AKI, RRT requirement, and death according to the Wnt5a levels in patients with urosepsis

**Figure 3.**
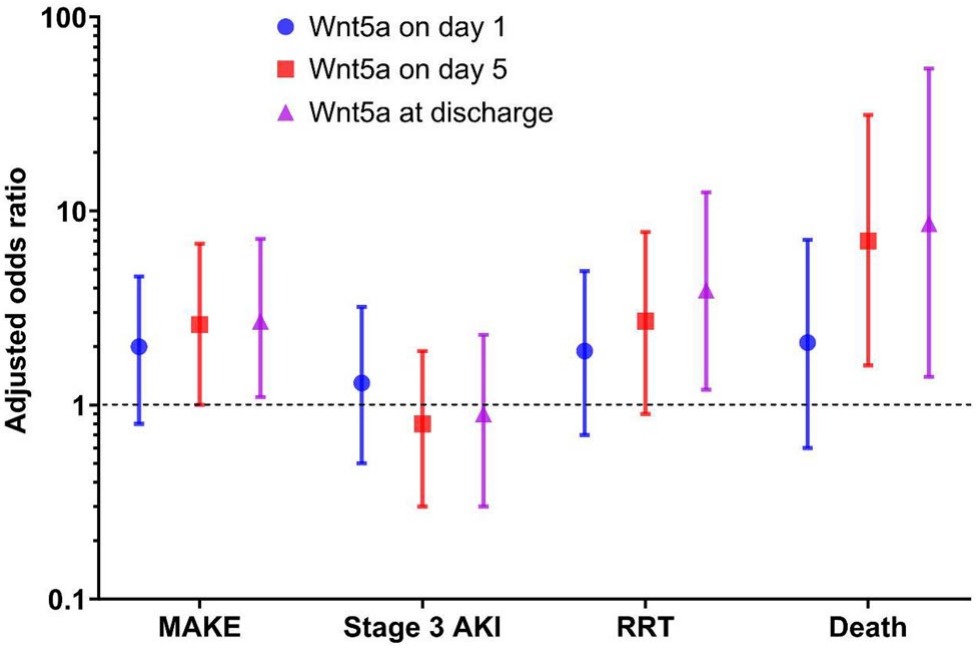
The adjusted likelihoods of Wnt5a for occurrence of MAKE and component in patients with urosepsis. The levels of Wnt5a seemed to be associated with MAKE (OR 2.0 [95% CI 0.8–4.6] on day 1, 2.6 [95% CI 1.0–6.8] on day 5, and 2.7 [95% CI 1.1–7.2] at discharge, respectively; *p* = 0.119, 0.060, and 0.039, respectively). Despite the lack of association between Wnt5a and stage 3 AKI (*p* = 0.557, 0.557, and 0.772, respectively), relationships were observed of Wnt5a with RRT (OR 1.9 [95% CI 0.7–4.9] on day 1, 2.7 [95% CI 0.9–7.8] on day 5, and 3.9 [95% CI 1.2–12.5] at discharge; *p* = 0.197, 0.067, and 0.020, respectively), and death (OR 2.1 [95% CI 0.6–7.1] on day 1, 7.0 [95% CI 1.6–31.3] on day 5, and 8.6 [95% CI 1.4–54.2] at discharge; *p* = 0.245, 0.010, and 0.022, respectively). CI: confidence interval; MAKE: major adverse kidney event; OR: odds ratio; RRT: renal replacement therapy. Data are presented as OR (95% CI). MAKE refers to new renal replacement therapy, stage 3 AKI, or death. The multivariate analysis included age, sex, baseline serum creatinine level, and sequential organ failure assessment scores for adjustment.

Among 87 patients with urosepsis, MAKE, stage 3 AKI, RRT requirement, and death occurred in 42 (48.3%), 31 (35.6%), 19 (21.8%), and 11 (12.6%) patients, respectively. The probability of MAKE and each of its components was estimated according to Wnt5a levels, which was expressed as the OR calculated using the logistic regression analysis (Figure 3). We found that elevated Wnt5a levels were associated with an increased risk of MAKE, especially at discharge. Among the components, the association between Wnt5a levels, RRT, and death was observed; however, Wnt5a levels were not associated with stage 3 AKI.

**Figure 4.**
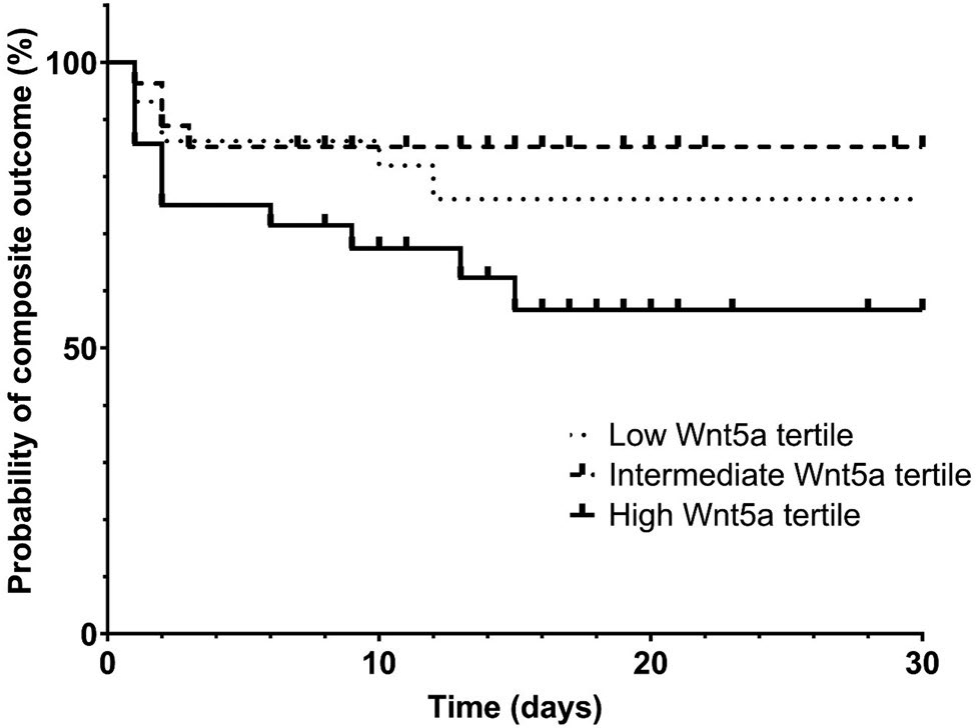
Cumulative incidence of renal replacement therapy requirement or death over 30 days according to initial Wnt5a levels. Patients with urosepsis were divided into the following tertiles: 29 (33.3%) in the low Wnt5a level, 29 (33.3%) in the intermediate tertile, and 29 (33.3%) in the high tertile. The cumulative incidence was higher in the high Wnt5a expression tertile than in the other tertiles (*p* = 0.097). The 30-day survival rates in the low, medium, and high tertiles were 76.0%, 79.3%, and 54.7%, respectively. RRT: renal replacement therapy. Composite outcome includes renal replacement therapy start and death.

Additionally, the time to the composite outcome of new RRT or death was evaluated according to the initial Wnt5a level by dividing into tertiles according to their day-1 Wnt5a levels. The cumulative incidence was higher in the high Wnt5a tertile than in the intermediate or low tertiles (Figure 4). In the multivariate Cox regression analysis, Wnt5a measurements predicted the occurrence of composite outcomes, especially those obtained on day 5 and the day of discharge (Table 3).

**Table 3.** The HRs of Wnt5a for new renal replacement therapy or death within 30 days in patients with urosepsis.

	Univariate		Multivariate^a^	
Variable	HR (95% CI)	*P*	HR (95% CI)	*P*
Wnt5a on day1	1.8 (0.9–3.3)	0.090	1.8 (0.9–3.7)	0.109
Wnt5a on day 5	2.7 (1.5–4.8)	0.001	2.5 (1.4–4.5)	0.003
Wnt5a at discharge	3.1 (1.5–6.5)	0.003	3.2 (1.4–7.1)	0.004

CI: confidence interval; HR: hazard ratio.

^a^ Adjusted for age, sex, baseline serum creatinine level, and sequential organ failure assessment score.

The influence of Wnt5a levels on the number of days free from RRT and ICU over 30 days was assessed using the multivariate linear regression analysis. There were trends toward fewer RRT-free days as Wnt5a levels increased (−1.9; 95% confidence interval [CI] − 4.3 to 0.5 on day 1, −1.7 [95% CI −4.2 to 0.8] on day 5, and −3.6 [95% CI −6.2 to −1.1] at discharge; *p* = 0.127, 0.175, and 0.006, respectively). Additionally, Wnt5a measurements were negatively associated with ICU-free days, irrespective of age, sex, baseline serum creatinine and SOFA score (−3.7 [95% CI −5.9 to −1.5] on day 1, −4.2 [95% CI −6.4 to −2.0] on day 5, and −5.0 [95% CI −7.4 to −2.7] at discharge; *p* = 0.001, *p* < 0.001, and *p* < 0.001, respectively).

### AKI recovery according to Wnt5a levels

**Figure 5.**
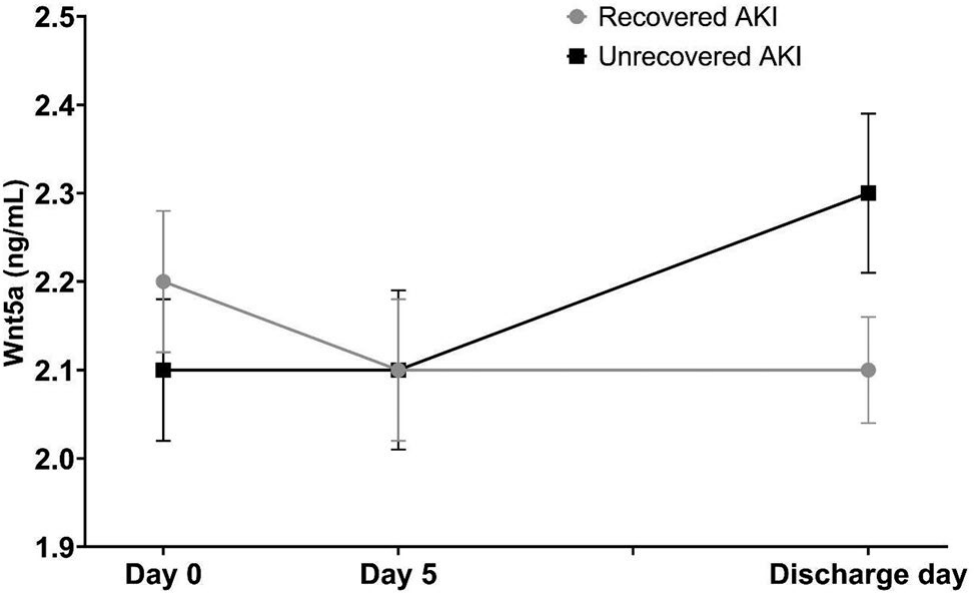
Wnt5a Levels during the study period according to the recovery pattern. The levels of Wnt5a were comparable between patients with recovered and unrecovered AKI (*p* = 0.596 on day 1, 0.486 on day 5, and 0.157 at discharge). However, the slope of Wnt5a over time differed between patients with and without recovery (P <0.001) and the declining rate of Wnt5a was steeper by −0.01 (95% CI −0.02 to −0.01) ng/mL per day in patients with recovered AKI than those without recovery. AKI: acute kidney injury; CI: confidence interval. Data are presented as mean ± standard error.

Of the included patients, 53 (60.9%) recovered from AKI, 34 (39.1%) did not recover, and 11 (12.6%) died. Wnt5a levels were comparable between patients with and without AKI recovery (*p* = 0.596 on day 1, 0.486 on day 5, and 0.157 at discharge; Figure 5). However, a trend for an association between discharge levels of Wnt5a and recovery was observed in the multivariate logistic regression analysis (OR 0.4 [95% CI 0.2–1.0]; *p* = 0.059). The Wnt5a levels on days 1 and 5 were not associated with AKI recovery (*p* = 0.295 and 0.434, respectively). The comparison of the trajectory of Wnt5a over time revealed that those with recovered AKI had a more negative slope than those with non-recovered AKI, independent of age, sex, baseline serum creatinine level, and SOFA score (*p* < 0.001; Figure 5).

## Discussion

This prospective observational study investigated the role of Wnt5a in AKI persistence in patients with urosepsis. Wnt5a levels remained consistently higher in patients with persistent or worsening AKI than in those with improving AKI; thus, this measurement seemed to be helpful in predicting the persistence of AKI in patients with urosepsis. Additionally, low Wnt5a levels and a declining slope appeared to predict AKI recovery. This study further evaluated the incidence of MAKE and revealed a relationship among Wnt5a levels, RRT requirement, and death.

AKI is a common complication in critically ill patients, particularly in those with sepsis [2]. In addition to the severity, the AKI duration and reversal pattern have been shown to influence short- and long-term outcomes [4,5]. A previous observational study described five AKI recovery patterns and reported that a prolonged AKI duration (>48 h) was associated with higher mortality and morbidity [5]. Additional research has confirmed the clinical importance of AKI duration on the prognosis in different settings [21–23]. Therefore, early identification of patients at risk of persistent AKI is warranted to promote preventive measures and improve patient outcomes; hence, novel bio-markers that reflect continuous inflammation and prolonged organ dysfunction are required. Moreover, AKI duration is crucial, since it is closely associated with an increased risk of chronic and end-stage kidney disease [7]. This study aimed to determine the clinical utility of predicting comprehensive kidney outcomes in patients with urosepsis by assessing Wnt5a expression and focusing on the persistence of AKI using serial Wnt5a measurements. Consistent with previous reports, this study confirmed that patients with persistent or worsening AKI had poor in-hospital outcomes, such as mortality and duration of RRT and ICU stay.

The ADQI Expert Group recently proposed the use of bio-markers in the prevention and management of AKI as well as to predict its duration and recovery [24]. However, gaps in knowledge and practice exist due to a lack of research. Some bio-markers appear to have potential despite the different definitions of persistent AKI. Plasma proenkephalin-A, urinary dickkopf-2, and urinary C–C motif chemokine ligand 14 have been evaluated in patients with sepsis and septic AKI [25,26], in those undergoing cardiac surgery [27], and in critically ill patients with AKI [28], respectively. In addition to these bio-markers, Wnt5a expression also appears to play a role in determining AKI persistence in patients with urosepsis. Notably, even day-1 levels predicted the persistence of AKI. Additionally, we observed a negative effect of Wnt5 levels on RRT- and ICU-free durations. Given the emerging evidence of the relationship between Wnt5a and inflammation, the clinical relevance of Wnt5a measurement and its relationship with disease severity and short-term outcomes has been investigated in various inflammatory conditions [15,16,29–31]. The results of the present study add to our knowledge of the possible interplay between Wnt5a and duration of organ dysfunction. Further research is required to understand the distinct pathophysiological mechanisms of the non-canonical pathway during the inflammatory process and to verify the role of Wnt5a as a novel bio-marker in populations with inflammatory disorders, such as sepsis.

This study evaluated the incidence of MAKE, including stage 3 AKI, initiation of RRT, and 30-day death, in patients with urosepsis, and revealed that Wnt5a levels were associated with RRT and death but not with stage 3 AKI. We previously reported a link between high Wnt5a levels and an increased occurrence of MAKE [16]; furthermore, this study illustrated detailed information by analyzing the MAKE components. The clinical significance of Wnt5a expression in sepsis has also been studied [14,15], and Schulte et al. [15] reported that Wnt5a levels declined within 5 days in patients recovering from sepsis, while the levels were maintained in patients without improvement. Moreover, the utility of Wnt5a in predicting the severity and progression of other inflammatory diseases, such as rheumatoid arthritis, systemic lupus erythematosus, and coronavirus disease-2019, has been evaluated [29–31]. Taken together, Wnt5a levels may be valuable for estimating the severity and prognosis of patients with acute or chronic inflammation. Based on our findings, follow-up values appeared to be more predictive; thus, serial measurements can be suggested for the management of such inflammatory diseases.

This study evaluated whether Wnt5a levels influence recovery and transition to acute and chronic kidney disease after AKI. In this study, the levels and trajectory of Wnt5a over time seemed to reflect the reversibility of AKI in patients with urosepsis. However, caution is necessary when interpreting our findings regarding the reversal pattern, as they were estimated using measurements obtained on the discharge day, which were not collected at the same time point. Both canonical and non-canonical Wnt pathways have been shown to have pivotal roles in recovery after AKI [32,33]. A study performed by Feng et al. [33] found that Wnt5a promotes kidney fibrosis by stimulating transforming growth factor-β-mediated macrophage polarization. Additionally, Wnt5a activation has been reported in several chronic diseases characterized by organ fibrosis [34–36]. Wnt5a can activate either canonical or non-canonical pathways, depending on spatio-temporal parameters and receptor availability [37]. Thus, further studies with serial measurements and histologic findings are required to confirm the role of Wnt5a in recovery after AKI.

This study had some limitations. First, it was performed at a single center with a small sample size, which could limit the statistical power and the detection of some differences. Nevertheless, this study recruited a homogenous sample of participants diagnosed with urosepsis; hence, it might enhance the clinical relevance by reducing bias caused by the multi-factorial nature of AKI in patients admitted to the ICU. Second, baseline serum creatinine levels were unavailable for a considerable proportion of patients (52.9%). We estimated baseline serum creatinine in these patients by back-calculation from the reference eGFR for age and sex using the Modification of Diet in Renal Disease equation, which was shown to be a preferred alternative [38]. We opted not to use values after admission as we had intended to evaluate renal recovery and to avoid using the assumed eGFR of 75 mL/min/1.73 m^2^ as we were concerned with the overestimation of AKI incidence in the elderly participants. Indeed, AKI and persistent or worsening AKI incidences were increased when assuming an eGFR of 75 mL/min/1.73 m^2^ (75.9% vs 71.3% and 35.6% vs 33.3%, respectively). Although our analysis revealed that the impacts of Wnt5a on outcomes did not differ between the two estimation methods, bias may have occurred as AKI incidence and outcomes tend to be affected by the methods to determine baseline creatinine [39]. Third, additional data, such as bio-markers of damage severity and reserve, and/or imaging and histological information, could have provided a better understanding of the pathophysiological mechanism in terms of duration and recovery after AKI.

In conclusion, our results indicated that elevated Wnt5a levels were associated with an increased risk of persistent AKI and a decreased probability of recovery. This study further observed the relationship between Wnt5a, RRT initiation, and death within 30 days in this disease population. Therefore, we suggest that Wnt5a levels can be a prognostic bio-marker in patients with sepsis and that serial measurements of this protein can help identify high-risk patients with persistent AKI and adverse clinical outcomes. Further studies with larger numbers of subjects are needed to understand the spatio-temporal role of Wnt5a throughout the disease and to verify Wnt5a measurements as bio-markers in various inflammatory disease conditions, including sepsis.

## Data Availability

The datasets generated during and/or analyzed in the current study are available from the corresponding author on reasonable request.
